# Green Chitosan Bioplastics: How the Filler Impacts the Biological Activity and the Biodegradability?

**DOI:** 10.3390/ma19102167

**Published:** 2026-05-21

**Authors:** Natalia Wrońska, Mohamed Amine Benzaouia, Beata Bielska, Agata Majkut, Maria Bryszewska, Katarzyna Miłowska, Abdelkrim El Kadib, Katarzyna Lisowska

**Affiliations:** 1 Department of Industrial Microbiology and Biotechnology, Faculty of Biology and Environmental Protection, University of Lodz, 12/16 Banacha Street, 90-236 Lodz, Poland; katarzyna.lisowska@biol.uni.lodz.pl; 2 Euromed Research Center, Engineering Division, Euromed University of Fès (UEMF), Route de Meknès, Rond-Point de Bensouda, Fès 30070, Morocco; m.benzaouia@ueuromed.org (M.A.B.); a.elkadib@ueuromed.org (A.E.K.); 3 Department of General Biophysics, Faculty of Biology and Environmental Protection, University of Lodz, 90-236 Lodz, Polandagata.majkut@edu.uni.lodz.pl (A.M.); maria.bryszewska@biol.uni.lodz.pl (M.B.); katarzyna.milowska@biol.uni.lodz.pl (K.M.); 4 Doctoral School of Exact and Natural Sciences, University of Lodz, 90-237 Lodz, Poland

**Keywords:** sustainable nanocomposites, chitosan films, nanosized filler, biological activity, biodegradability

## Abstract

**Highlights:**

- Chitosan presents a biodegradable alternative to synthetic plastic.
- Filler incorporation enhanced the functional properties of chitosan films.
- Biodegradable chitosan nanocomposites with antibacterial activity and low cytotoxicity.

**Abstract:**

The growing environmental plastic pollution triggered research for biodegradable and safe materials, among which biopolymer-based films stand as the most promising. Among these, chitosan has gained significant attention due to its biocompatibility, film-forming ability, and inherent antimicrobial properties. In this context, the use of fillers to design chitosan nanocomposite films has been shown to enhance the mechanical, barrier, thermal, optical, and antimicrobial properties of the resulting bioplastics. However, the fate and destiny of these fillers, as well as their impact on the biological properties and biodegradability of chitosan films, remain underexplored. We herein report a more comprehensive screening of a set of fillers, encompassing three clay variants (montmorillonite, sepiolite, and halloysite) and microcrystalline chitin. The films were systematically characterized to assess their antibacterial performance, cytocompatibility, hemocompatibility, and biodegradability. The highest antibacterial activity was observed for *CS@MMT-f* film towards *Staphylococcus aureus* and *Escherichia coli*. Importantly, all developed films demonstrated negligible hemolytic activity and low cytotoxicity, indicating their safety for potential biomedical or food-contact applications. Moreover, the selected films completely degrade within four to six weeks under soil burial conditions, demonstrating their potential as environmentally friendly packaging materials.

## 1. Introduction

In recent years, biopolymers have garnered increasing attention as a sustainable alternative to synthetic polymers, driven by growing environmental concerns and the urgent need to limit plastic waste generation. Petroleum-based plastics suffer from slow degradation, and even worse, their disintegration leads to the so-called micro- and nanoplastics, two variants that penetrate human bodies and the surrounding environment [1]. An ideal plastic requires at least 90% disintegration after twelve weeks, and 90% biodegradation involving CO_2_ in only six months, according to the European bioplastics industry standards for biodegradable plastics. Biopolymers, which are extracted from renewable biological sources such as plants, animals, and microorganisms, offer several advantages over petroleum-based plastics, including sustainability, availability, biodegradability, and a lower carbon footprint [2,3]. Biopolymer-based materials include polysaccharides (cellulose, chitosan, starch, etc.), proteins (soy protein, casein, gelatin, etc.) and lipids (beeswax, carnauba wax, etc.) [4,5,6,7]. From a technological standpoint (low cost, easy acquisition and modification), chitosan is an excellent biopolymer, which, due to its biological properties, can be used in various industries [8]. Chitosan (CS) is derived from chitin, which, combined with calcium carbonate, constitutes the exoskeletons of crustaceans such as shrimp, crabs, and lobsters [9,10]. It is produced through the deacetylation of chitin and is mostly composed of (1–4)-linked 2-amino-2-deoxy-β-D-glucose monomers. Chitosan and its derivatives have gained considerable interest in various fields, including biomedical applications [11,12,13], food packaging [14,15,16], agriculture [17,18], water treatment [19,20], and the pharmaceutical industry [21,22,23]. Despite its many beneficial properties, the practical applications of chitosan are limited. Its poor solubility in neutral and alkaline pH environments limits its use in some biological and industrial processes [24]. Furthermore, chitosan’s mechanical strength and thermal stability are relatively low compared to synthetic polymers. It has been shown that CS films can be chemically modified, blended with other materials, or reinforced with functional compounds to enhance their chemical and thermal stability, mechanical properties, and even biological activity [25,26,27,28,29,30]. Research indicates that carbon-based materials, metal (oxide) nanoparticles, clay nanoparticles, bio-ceramics, and other well-shaped particulates are excellent reinforcements for nanocomposites in a chitosan matrix. More recently, bio-based fillers derived from natural resources have also emerged as greener alternatives, thereby expanding the library of the crafted bioplastics towards more sustainable variants [31]. Different type of fillers, the origin of their reinforcing properties, and their mechanisms of action are gathered in Supplementary Table S1 to provide an overview of their use in the field of bioplastic coating and packaging. Schnabl et al. (2024) investigated the effect of a set of chemical additives, commonly used in the plastic industry, on chitosan films, with a special emphasis on their long thermal stability, weight loss, and film disintegration depending on aging and storing conditions [32]. This study concluded on the excellent properties of citric acid, and to a lesser extent, lactic acid for conceiving practical chitosan-based films [32]. Comparatively, few related data are available concerning the impact of the fillers, where it is not yet clear whether they compromise the biocompatibility of the conceived nanocomposite films and to what extent they could hinder or delay their biodegradability. Given the importance of adding both fillers and plasticizers, among other functionalities, gathering complementary knowledge in the two directions could indeed help promoting the use of chitosan in the realm of the plastic industry.

Clay materials are used because of their large specific surface area, ion exchange capacity and layered charges [33]. Moreover, their excellent mechanical and rheological properties give them a wide range of applications as auxiliary substances to improve technological properties, e.g., in the packaging industry. In pharmacy, clays are used in drug delivery systems. They can increase the solubility of the drug, control its release and prevent side effects [34]. They are added as reinforcement to green composites in which the matrix and/or reinforcement are made from biodegradable and renewable resources [35].

The purpose of this work was to comprehensively evaluate biopolymer-based composite films containing selected natural fillers, including montmorillonite, sepiolite, halloysite, and microcrystalline chitin, with particular emphasis on the influence of the filler type on the biological and environmental properties of the materials. The study aimed to assess the antibacterial activity, cytocompatibility, and hemocompatibility of the developed composites, as well as to determine how the incorporation of different fillers affects the biodegradation behavior of the films in a soil environment.

## 2. Materials and Methods

### 2.1. Materials

Chitosan of medium molecular weight and 85% deacetylation degree was purchased from Sigma-Aldrich (Hamburg, Germany). Microcrystalline chitin was provided as a dry powder by France Chitine (Orange, France). Cellulose nanocrystals (L_NCC_ = 130 50 nm; D_NCC_ = 25 15 nm) were prepared from microcrystalline cellulose in H_2_SO_4_ (65 wt%) at 318 K for 60 min, following procedures described elsewhere [36]. Montmorillonite (denoted herein as MMT) was purchased from Alfa Aesar (Karlsruhe, Germany). Sepiolite is built from slightly aggregated fibers with a length of a few micrometers. Halloysite nanotubes (high purity: 99%; 1% of Fe_2_O_3_ and TiO_2_ and CaCO_3_ impurities), referred herein as HNT, with the following parameters (length: 0.2–2 m; outside diameter 50–70 nm; inside diameter 15–45 nm) was made available by Dragonite company (Eureka, UT, USA). Human erythrocytes were obtained from buffy coats provided by the Regional Blood Donation and Blood Treatment Center in Lodz.

The human skin fibroblast cell line BJ (CRL-2522) was purchased from the American Type Culture Collection ATCC (Manassas, VA, USA). Reagents used for cell culture, such as Dulbecco’s Modified Eagle Medium (DMEM), fetal bovine serum (FBS), and antibiotics (streptomycin and penicillin), were from Gibco, a Thermo Fisher Scientific company (Waltham, MA, USA). Dimethylsulfoxide (DMSO), phosphate-buffered saline (PBS) tablets, trypan blue and trypsin were supplied by Sigma-Aldrich (Saint Louis, MO, USA).

### 2.2. Preparation of Pristine and Filled Chitosan Bioplastic Films

**Pristine chitosan film.** An amount of 50 mg of chitosan was solubilized in 4 mL of acidified water (2 mL of acetic acid in 98 mL of water) for 4 h. The resulting colloidal gelling solution was poured into Petri dishes, resulting, after solvent evaporation, on pristine chitosan film.

**CS@ChitMC film.** Chitosan in the amount of 50 mg was solubilized in 4 mL acidified water (2 mL of acetic acid in 98 mL of water) for 4 h until the formation of the gel. A total of 1.5 mg of chitin micro crystals was dispersed in 2 mL of 1% (*v*/*v*) aqueous acetic acid solution. The filler dispersion was gradually added to the CS solution, and the resulting mixture was stirred for further 4 h. The resulting solution was cast into plastic Petri dishes, allowing solvent removal and film formation after drying.

**CS@MMT film.** A total of 50 mg of chitosan was solubilized in 4 mL acidified water (2 mL of acetic acid in 98 mL of water) for 4 h until the formation of the gel. Then, 1.5 mg of MMT, previously dispersed in 2 mL of water, was added. The mixture was stirred for 4 h at room temperature and then introduced into a Petri dish under static conditions. Upon water removal and gel drying, good quality, easy-to-handle, crack-free films were obtained.

**CS@Sep film.** 50 mg of chitosan was solubilized in 4 mL acidified water (2 mL of acetic acid in 98 mL of water) for 4 h until the formation of the gel. Then, 1.5 mg of Sepiolite, previously dispersed in 2 mL of water, was added. The mixture was stirred during 4 h at room temperature and then introduced in a Petri dish under static conditions. Upon water removal and gel drying, good quality, easy-to-handle, crack-free films were obtained.

**CS@HNT film.** A total of 50 mg of chitosan was solubilized in 4 mL acidified water (2 mL of acetic acid in 98 mL of water) for 4 h until the formation of the gel. Then, 1.5 mg of HNT previously dispersed in 2 mL of water was added. The mixture was stirred during 4 h at room temperature and then introduced in a Petri dish under static conditions. Upon water removal and gel drying, good quality, easy-to-handle, crack-free films were obtained.

### 2.3. Determination of Antimicrobial Activity

The antimicrobial activity of chitosan nanocomposite films against Gram-positive bacteria: *Staphylococcus aureus* ATCC 6538, and Gram-negative bacteria: *Escherichia coli* ATCC 25922 was evaluated using the Japanese Industrial Standards JIS Z 2801:2000, with modification. The bacterial strains were cultured on Luria–Bertani (LB) medium at 37 °C on a rotary shaker. After the incubation, the inoculum of tested bacteria containing 1 × 10^5^ colony-forming units (CFU per mL) in 500-fold diluted LB medium was prepared. Next, the suspension of bacteria was applied to chitosan films of 2 cm × 2 cm. Native chitosan films were analyzed as control samples. After dripping the suspension of the tested bacterial strains on the films, each sample was covered with a sterile film (1.7 cm × 1.7 cm). The samples were incubated in the moist chamber in the dark for 24 h at 37 °C. After incubation, the samples were put in aseptic Falcon tubes containing phosphate buffer and vortexed. Next, samples were removed from the Falcon tubes, and the remaining solution was used to perform a serial dilution in phosphate buffer. Out of each dilution, 100 µL of bacterial suspension was seeded on an agar plate and incubated for 24 h at 37 °C. After incubation, CFU (Colony-Forming Unit) was calculated by counting the visible colonies on a plate. Each type of tested nanocomposite films was examined in triplicate and analyzed individually in four independent experiments. The antimicrobial activity of the tested films was calculated as the percentage of bacterial growth inhibition (SD) towards the control (native chitosan film).

### 2.4. Hemolysis Assay

Red blood cells (RBCs) were separated from the buffy coat by centrifugation at 3000 rpm for 10 min at 4 °C. Following isolation, the erythrocytes were washed three times with phosphate-buffered saline (PBS, pH 7.4) to remove residual plasma and leukocytes. Experimental conditions were established by suspending erythrocytes at a hematocrit of 2% in the presence of square-shaped chitosan films (0.5 × 0.5 cm^2^). These preparations were incubated at 37 °C for 24 h.

Control samples consisted of erythrocytes suspended in PBS without the chitosan film, while a positive control for 100% of complete hemolysis was prepared by suspending erythrocytes in distilled water. After incubation, all samples were subjected to a centrifugation step under the same conditions (3000 rpm, 10 min). The supernatants were collected and transferred to a 96-well microplate for spectrophotometric analysis. Absorbance was measured at 540 nm using a BioTek Synergy HTX microplate reader (Winooski, VT, USA). Hemolysis was quantified by determining the concentration of hemoglobin released into the supernatant, using the following formula:

$$hemolysis\;\left[\%\right]=\frac{A_{s}}{A_{100\%\;of\;hemolysis}}\times 100\%$$

A_s_—absorbance of the sample

A_100% of hemolysis_—absorbance of the sample with water (100% of hemolysis).

### 2.5. Assessment of Hemoglobin Adsorption by Chitosan-Based Composites

To evaluate the potential of the tested chitosan-based composites to adsorb hemoglobin from the surrounding medium, square-shaped chitosan films (0.5 × 0.5 cm^2^) were immersed in hemoglobin solutions at a concentration of 0.1% *v*/*v*. The samples were incubated at 37 °C for 24 h to allow interaction between the film and the protein. Following incubation, the mixtures were centrifuged, and the residual hemoglobin concentration in the supernatant was determined spectrophotometrically at 540 nm. The percentage of hemoglobin adsorption was calculated based on the decrease in absorbance, using the following formula:

$$Adsorption\;of\;Hb\left[\%\right]=100\%-(\frac{A_{s}}{A_{c}}\times 100\%)$$

where

A_s_—absorbance of the sample containing the chitosan composites

A_c_—absorbance of the control (hemoglobin without chitosan composites).

### 2.6. Spectrophotometric Determination of Methemoglobin Content

The oxidation of hemoglobin to methemoglobin triggered by chitosan films was investigated using spectrophotometry. Erythrocyte suspensions were incubated with films (0.5 × 0.5 cm^2^) for 24 h at 37 °C.

The proportion of methemoglobin (metHb) relative to total hemoglobin (Hb) following 24 h incubation with the composite materials was calculated based on absorbance values at 630 nm and 700 nm. Hemoglobin oxidized with potassium ferricyanide, representing 100% metHb, was used as the positive control.

$$\%metHb=\frac{A_{630}-A_{700}}{A_{630}^{\#}-A_{700}^{\#}}\times 100\%$$

where

A_630_ i A_700_—absorbance of the sample at 630 and 700 nm

$A_{630}^{\#}$ i $A_{700}^{\#}$—absorbance of the sample with potassium ferricyanide at 630 and 700 nm.

### 2.7. Cell Culture

BJ cells were grown as a monolayer in DMEM supplemented with 10% fetal bovine serum (FBS) and 1% streptomycin. The cultures were incubated at 37 °C in an atmosphere of 5% CO_2_. Cells were split for subcultures every 2 days and were kept in log phase by regular passage according to the procedure described previously [37].

### 2.8. Cell Viability (MTT Test)

Cell viability was evaluated using the MTT assay following a 24 h exposure to chitosan-based nanocomposites. Cells were seeded at a density of 5 × 10^4^ per well into a 24-well culture plate and incubated overnight at 37 °C in a humidified atmosphere containing 5% CO_2_. The next day, the culture medium was replaced with fresh medium and chitosan nanocomposites were added to the samples.

After 24 h of incubation, the cells were washed with phosphate-buffered saline (PBS) and subsequently treated with MTT reagent (0.5 mg/mL; 3-(4,5-dimethylthiazol-2-yl)-2,5-diphenyl tetrazolium bromide) for 3 h at 37 °C. Following incubation, the MTT solution was removed, and dimethyl sulfoxide (DMSO) was added to solubilize the formazan crystals formed by metabolically active cells, as described by Mossman [38].

Absorbance was measured at 570 nm using a BioTek Synergy HT microplate reader (BioTek Instruments, Winooski, VT, USA). Cell viability was expressed as a percentage relative to the untreated control group, calculated using the following formula:

(1) $$Viability\;\left[\%\right]=\frac{A_{s}}{A_{c}}\times 100\%$$

where

A_s_—absorbance of the sample

A_c_—absorbance of the control (untreated cells).

### 2.9. Biodegradability of Chitosan Films in Soil

The study presents evaluating the biodegradability of chitosan films via determinating the degradability of the CS films by soil microorganisms. The soil samples were collected in the garden of the Faculty of Biology and Environmental Protection University of Lodz, Poland, and used as an incubation medium. The soil was maintained at room temperature, 45% moisture, and pH equal to 6. The soil was placed in a special container and sterile samples were buried at a depth of approximately 10 cm (each sample in a separate container) and incubated at room temperature. The buried samples were dug out at different periods of time (1, 2, 4 and 6 weeks of incubation) and rubbed gently with brush to remove soil residues. Then, the samples were rinsed thoroughly with distilled water, dried at 50 °C for 4 h, desiccated for 24 h at room temperature, weighted and the percent of weight loss of samples was determined. We analyzed 5 films: chitosan (CS-*f*), chitosan–chitin (CS@ChitMC-*f*), chitosan–MMT (CS@MMT-*f*), chitosan–sepiolite (CS@Sep-*f*), chitosan–HNT (CS@HNT-*f*). The obtained images of CS films were compared with images after 1-, 2-, or 4-week period incubation.

### 2.10. Statistical Analysis

Data are presented as the mean ± SD from a minimum of 3 sets of measurements. Statistical differences between the control and treatment groups were analyzed by one-way ANOVA followed by Tukey’s analysis. *p* < 0.05 was taken as statistically significant.

## 3. Results

### 3.1. Characterization of Filled Chitosan Nanocomposites

**Figure 1:**
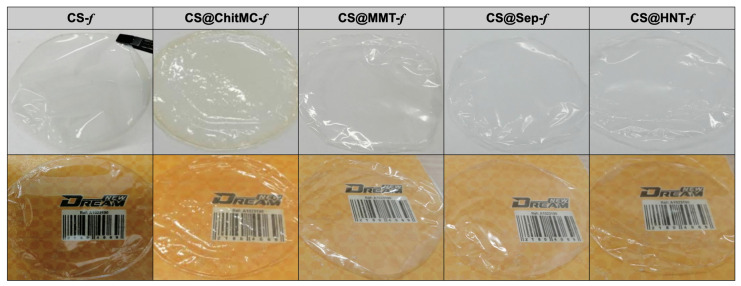
Images of the visual appearance of the chitosan (**CS-*f***), chitosan–chitin (**CS@ChitMC-*f***), chitosan–montmorillonite (**CS@MMT-*f***), chitosan–sepiolite (**CS@Sep-*f***), and chitosan–HNT (**CS@HNT-*f***).

Regardless of the nature of the filler, a homogenous chitosan aqueous acidic solution containing 3 wt% of dispersed filler was prepared. Evaporative-induced assembly of the resulting solutions provides high-quality, crack-free, flexible and easy-to-handle chitosan-based bioplastic films. Their photos are presented below along with pristine chitosan films prepared for comparison (Figure 1).

**Figure 2:**
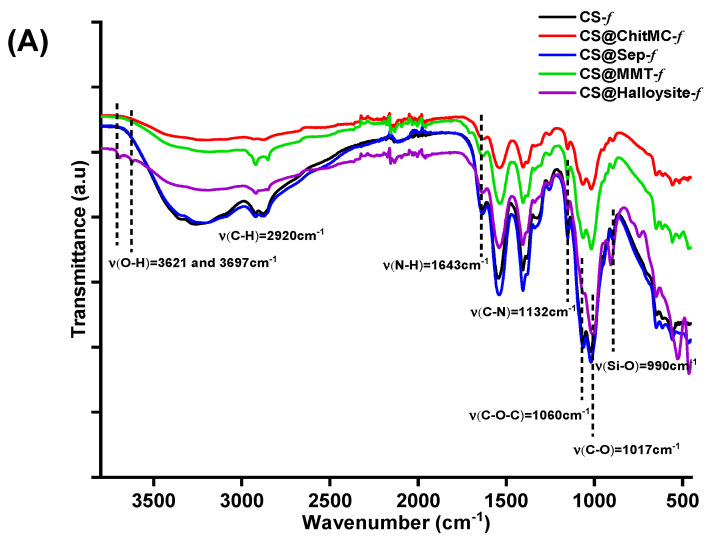

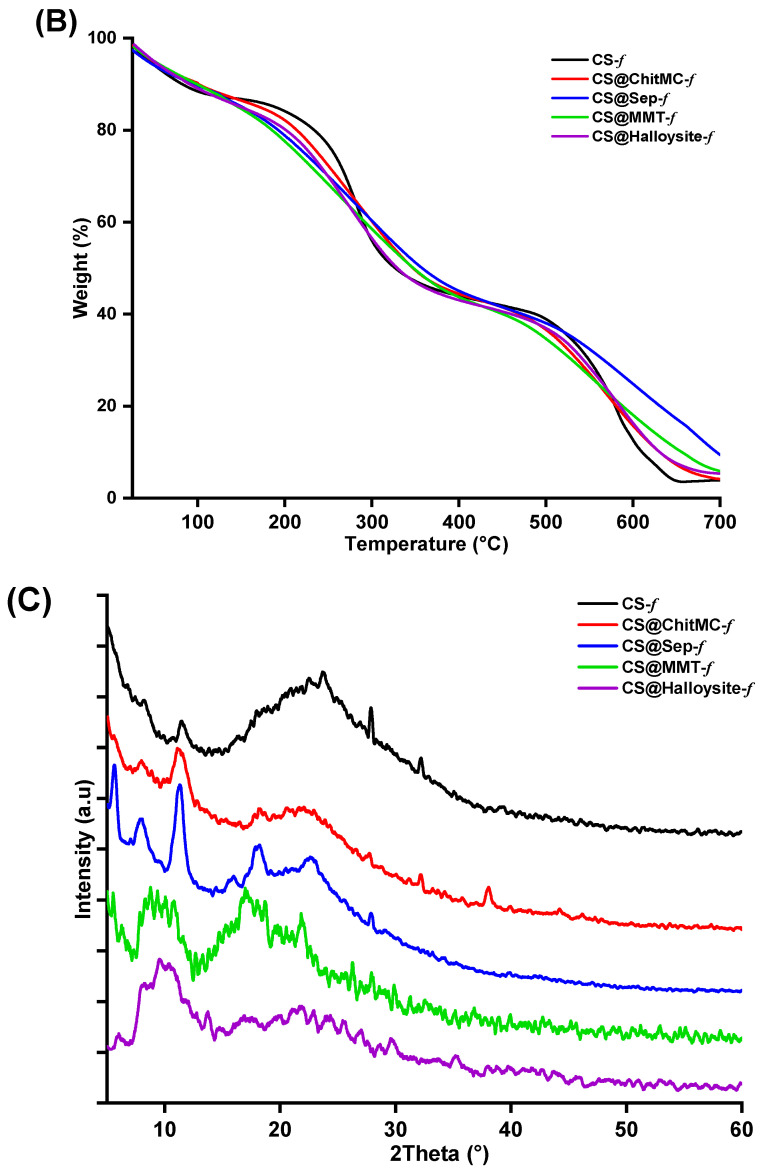
Characterization of chitosan nanocomposites: (**A**) FTIR analysis, (**B**) TGA profile of tested films, (**C**) XRD analysis.

The FTIR spectrum of the prepared films shows primarily the fingerprint signature of chitosan polymer, the latter being more abundant compared to 3 wt.% of the filler inserted (Figure 2A). The common bands attributed to C–O, C–O–C, C–N, N–H, acetamide C–H, and O–H vibrations appear at 1017, 1060, 1160, 1332, 1544, 1643, 2870, 2920, and 3200–3400 cm^−1^. The increase in the band around 990 cm^−1^, assigned to Si–O stretching, confirms the presence of clay minerals (montmorillonite and halloysite) within the chitosan matrix. Furthermore, two additional bands observed at 3621 and 3697 cm^−1^ are attributed to the hydroxyl groups located on the internal and external surfaces of halloysite nanotubes (HNTs), providing further evidence for the successful incorporation of clays into the chitosan films.

Thermogravimetric analysis reveals that unmodified chitosan film exhibits the highest thermal stability at lower temperatures (Figure 2B). However, this trend is reversed at higher temperatures. In particular, the chitosan film reinforced with sepiolite shows the highest char residue (10%) and the highest T_50_ value (360 °C), indicating improved thermal resistance due to the presence of the clay filler.

The X-ray diffraction (XRD) pattern of native chitosan films shows two characteristic peaks at 2θ ≈ 10° and 20°, corresponding to the semi-crystalline structure of chitosan (Figure 2C). After the incorporation of chitin (**CS@ChitMC-f**), a slight increase in peak intensity is observed, indicating the contribution of the crystalline domains of chitin within the chitosan matrix. The pattern of the composite films was significantly modified with the incorporation of clay particulates, indicating that secondary chitosan organization has been altered by the entanglement of the filler particulates within the polysaccharide network. This profile is indeed indicative of the occurrence of strong interactions between chitosan chains and dispersed clay particles, as it has been already reported for similar systems [39,40].

### 3.2. Biological Activity of Chitosan Nanocomposite Films

#### 3.2.1. Antimicrobial Activity

**Figure 3:**
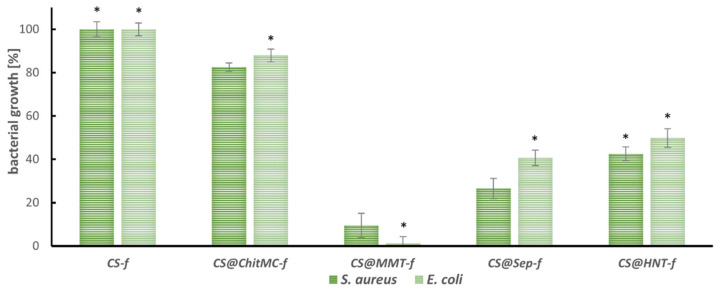
Antibacterial activity of the nanocomposite films, bacterial growth [%] after 24 h incubation (n = 6, * *p* < 0.05).

The antimicrobial activity of chitosan nanocomposite films: chitosan (**CS-*f***), chitosan–chitin (**CS@ChitMC-*f***), chitosan–montmorillonite (**CS@MMT-*f***), chitosan–sepiolite (**CS@Sep-*f***), chitosan–nanotubular halloysite (**CS@HNT-*f***), was assessed using *Staphylococcus aureus* ATCC 6538 and *Escherichia coli* ATCC 25922 as model bacteria and taking native chitosan film CS-*f* as a reference (Figure 3).

All filler-containing chitosan nanocomposites limited the growth of the tested microorganisms compared to the native chitosan coating. In the case of the combination of chitosan and chitin (**CS@ChitMC-*f***), the antibacterial activity was low. Only 10–17% growth inhibition of the tested strains was observed. The highest antibacterial activity was observed for the CS-MMT (**CS@MMT-*f***) film. The chitosan-reinforced montmorillonite nanocomposites caused almost 100% inhibition of *S. aureus* growth and 91% inhibition of *E. coli*. In the case of the other two composites enriched with clay minerals, chitosan–sepiolite showed better activity against the tested bacteria (70% inhibition of *S. aureus* growth) compared to CS–halloysite (**CS@HNT-*f***). The addition of sepiolite or halloysite demonstrated lower growth inhibition of the Gram-negative *E. coli* strain by 60% and 50%, respectively.

#### 3.2.2. Hemolysis and Hb Adsorption

**Figure 4:**
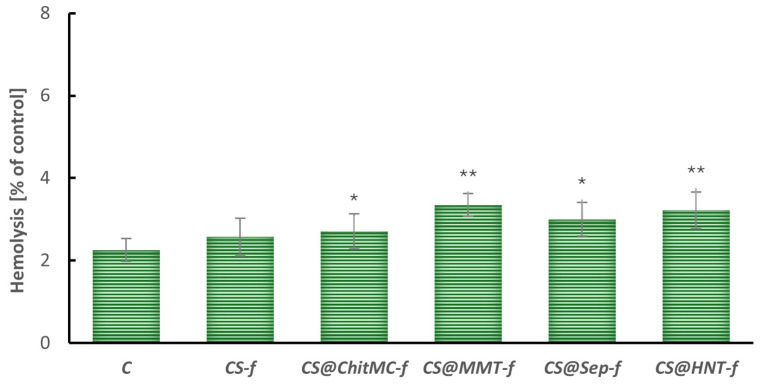
Hemolysis of human red blood cells after incubation (24 h) with chitosan-modified films (n = 6, * *p* < 0.05, ** *p* < 0.01).

The hemolysis test results, presented in Figure 4, indicate a statistically significant increase in the degree of erythrocyte membrane damage in the tested chitosan-based materials (excluding unmodified chitosan). The control sample (C) showed a baseline level of hemolysis of approximately 2%, providing a reference point for assessing the biocompatibility of the modified films. It was demonstrated that despite the statistically significant changes, the increase in hemolysis was small, and the percentage of hemolysis after 24 h of incubation with the nanocomposites was below 5%.

Previous studies have demonstrated that chitosan-based materials possess the ability to adsorb hemoglobin onto their surface, potentially influencing the accuracy of hemolysis measurements [41,42].

**Figure 5:**
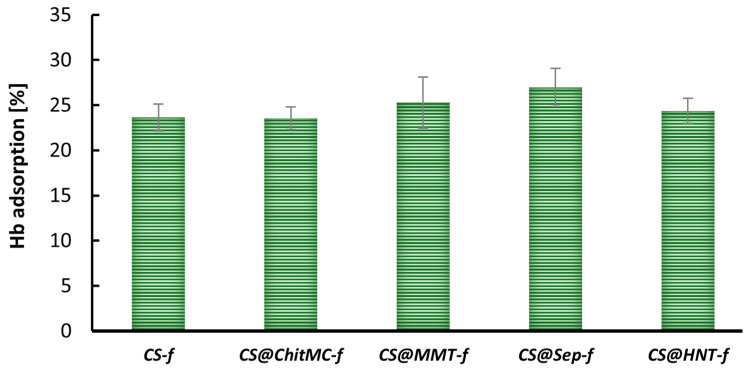
Adsorption of hemoglobin to chitosan films (n = 6, *p* < 0.05).

In the present study, the hemoglobin adsorption capacity of various chitosan-based films was also evaluated (Figure 5). As illustrated in Figure 5, the unmodified chitosan film (**CS-*f***) adsorbed approximately 25% of the available hemoglobin, serving as a reference for comparative analysis across the tested materials.

Most of the materials tested exhibited Hb adsorption levels ranging from approximately 20% to 30%, indicating moderate affinity for hemoglobin. Among the samples, **CS@Sep-*f*** demonstrated the highest adsorption capacity, with values slightly exceeding 30%, suggesting a strong interaction between the film surface and hemoglobin molecules.

#### 3.2.3. Methemoglobin

**Figure 6:**
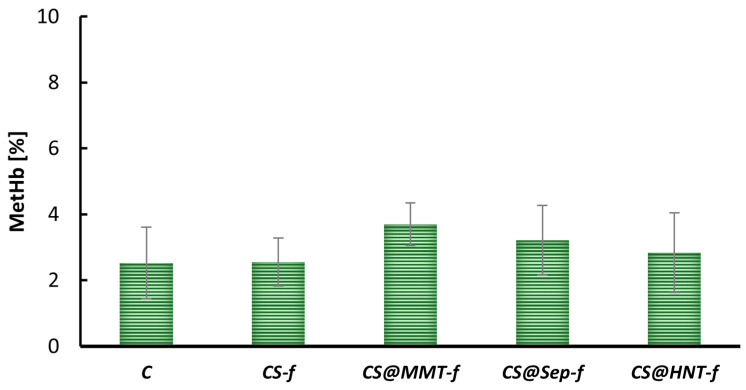
Methemoglobin content in human erythrocytes exposed to chitosan films after 24 h of incubation at 37 °C (n = 6).

Methemoglobin (MetHb) formation was assessed to evaluate the oxidative stress induced by various chitosan-based films. As shown in Figure 6, the control sample (C) had a baseline MetHb level of approximately 2.5%. All tested materials had MetHb levels similar to the control, with values ranging from approximately 2.5% to 4%.

These results indicate that the chemical modifications used do not cause oxidative stress in erythrocytes, as no significant changes in MetHb levels were observed compared to the control.

#### 3.2.4. Cytotoxicity

**Figure 7:**
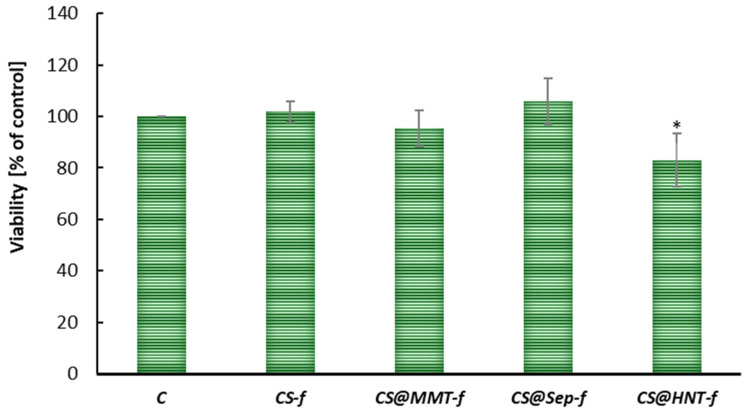
Viability of human skin fibroblasts (BJ) treated with chitosan films for 24 h at 37 °C (n = 6, * *p* < 0.05).

The cytotoxicity of chitosan-based films was assessed by analyzing the viability of human skin fibroblasts (BJ) after 24 h of exposure. As shown in Figure 7, the control group (C) was designated as 100% viability, serving as a reference for untreated cells.

Most tested films maintained cell viability close to or above the control level, indicating good biocompatibility. The samples **CS-*f***, **CS@ChitMC-*f***, **CS@MMT-*f***, **CS@Sep-*f*** showed viability levels ranging from approximately 90% to 106%, suggesting that these formulations do not exert cytotoxic effects and may even promote cell proliferation in some cases.

In contrast, sample **CS@HNT-*f*** demonstrated a statistically significant reduction in cell viability (*p* < 0.05), indicating potential cytotoxicity or adverse interactions with fibroblast cells. However, the observed reduction in cell viability is relatively minor, not exceeding 20%, which suggests that the tested films can also be considered biocompatible with skin cells.

#### 3.2.5. Biodegradability of Chitosan Films in Soil

**Figure 8:**
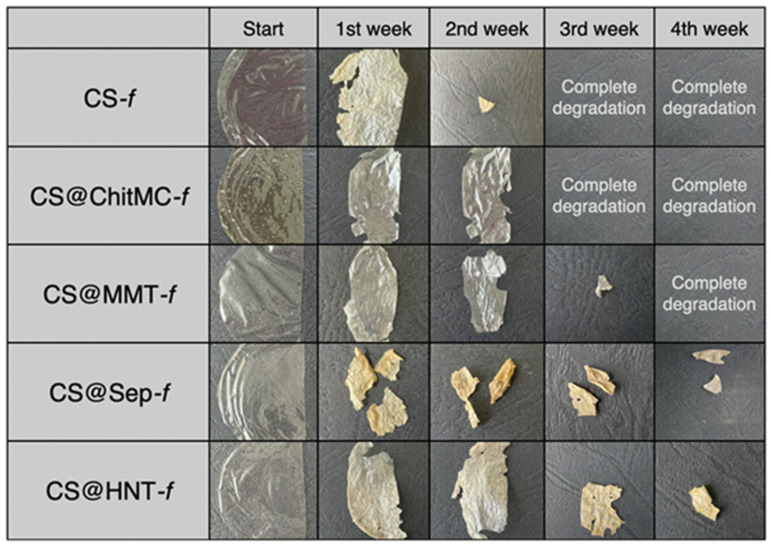
Images of the visual appearance of the chitosan (**CS-*f***), chitosan–chitin (**CS@ChitMC-*f***), chitosan–MMT (**CS@MMT-*f***), chitosan–sepiolite (**CS@Sep-*f***), chitosan–NT (**CS@HNT-*f***)—during incubation in the soil. Time incubation, t = 0, 1, 2, 3, 4 weeks.

All tested films were homogeneous and were stored at room temperature. Each film was halved and photographed before being placed in the soil. The biodegradation of these films in a soil environment was examined and compared with that of pure chitosan films. The photographs presented in Figure 8 document the results obtained during the incubation of nanocomposites in the soil environment. All tested films are presented in Table 1 (Section 2).

The biodegradation of those films was studied and compared with a pure chitosan film. The results were recorded with photographs, which are illustrated in Figure 7. In soil, placed in separate containers and incubated at room temperature, ***CS-f***, **CS@ChitMC-*f***, ***and*** **CS@MMT-*f*** were degraded after 4 weeks of incubation. The results clearly showed that chitosan modifications can significantly affect the decomposition of the tested materials.

After 2 weeks of incubation, almost complete degradation of the pure chitosan film (***CS-f***) was observed, along with a significant loss in the ***CS@Sep-f*** sample. The other films were wrinkled, pliable with a slight loss of composite mass (Figure 8). The greatest changes were noted after 3 weeks of incubation of the samples in the soil environment. Complete biodegradation of the ***CS-f***, **CS@ChitMC-*f*** and **CS@MMT-*f*** films occurred. Furthermore, a large mass loss was observed in the **CS@HNT-*f*** (Figure 8). After 4 weeks of incubation, complete biodegradation of **CS@MMT-*f*** was observed, while after 6 weeks, no **CS@Sep-*f*** or **CS@HNT-*f*** films were observed in the soil.

**Figure 9:**
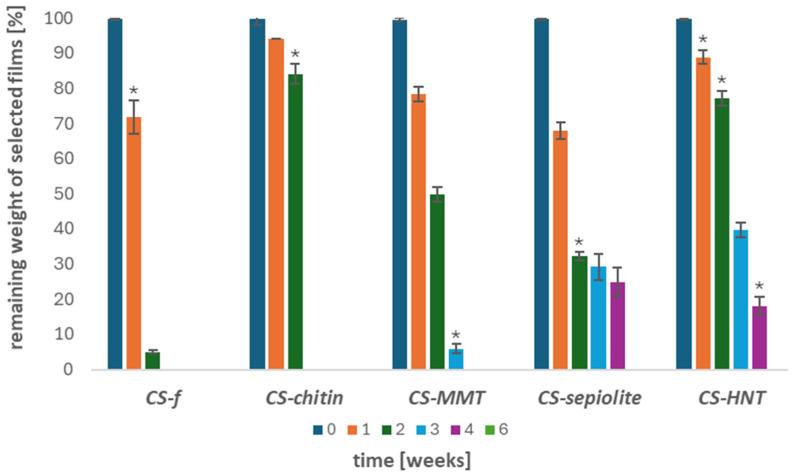
Change in the weight ratio in chitosan (**CS-*f***), chitosan–chitin (**CS@ChitMC-*f***), chitosan–MMT (**CS@MMT-*f***), chitosan–sepiolite (**CS@Sep-*f***), and chitosan–HNT (**CS@HNT-*f***) films exposed to the soil environment in 1, 2, 3, 4 and 6 weeks of incubation time (n = 3, * *p* < 0.05).

Moreover, the extent of weight loss of the tested materials was studied. The results are presented in Figure 9.

Figure 9 shows the changes in the weight ratio of the tested chitosan films over 1, 2, 3, 4, and 6 weeks of incubation. After the first week, approximately 30% weight loss was observed for both **CS-*f*** and ***CS@Sep-f*** films. By the second week, the **CS-*f*** film had nearly completely degraded, while the ***CS*@*MMT-f*** and ***CS@Sep-f*** samples exhibited significant weight loss. After 3 weeks of incubation, complete (100%) weight loss was observed for **CS-*f*** and **CS@ChitMC-*f***, whereas for **CS@MMT-*f*** this occurred after 4 weeks. The remaining samples reached total degradation after 6 weeks of incubation in the soil environment.

## 4. Discussion

The incorporation of mineral fillers into chitosan-based bioplastic films significantly modulates both their biological activity and biodegradability, largely through changes in structural organization, interfacial interactions, and physicochemical properties. In this study, the nature of the filler, including its chemical composition, morphology, and surface functional groups, emerged as a factor governing performance. ***CS@MMT-f*** composites exhibited superior antibacterial activity towards *Staphylococcus aureus* and *Escherichia coli*. In this case, almost complete inhibition of *E. coli* growth and 90% inhibition of *S. aureus* growth were observed. Similar results were obtained by Liu et al. (2025), demonstrating a broad spectrum of antimicrobial activity of the montmorillonite-chitosan hydrogel system, especially against *E. coli* and *S. aureus* [43]. In other studies, the CS-MMT composite was tested as an anticancer drug carrier. The incorporation of MMT into the CS matrix reduced the release rate of 5-FU by entrapping it within the clay layers, thereby enhancing the antibacterial activity of the CS–clay/drug nanocomposite film against *S. aureus* and *E. coli.* [44]. CS–sepiolite (***CS@Sep-f***) also showed promising antimicrobial effects, likely due to its fibrous structure and surface chemistry. Dutta et al. (2021) investigated that the CS-SEP films showed better antibacterial activity than chitosan alone against both *B. subtilis* and *E. coli* [45]. It was revealed that the addition of sepiolite to chitosan–silver composites, apart from enhancing its stability and antimicrobial activity, also allowed for a reduction in cytotoxicity (compared to silver alone). They report average widths of inhibition zones (disk diffusion) of ≈51.8 mm against *S. aureus*, ≈31.8 mm vs. *E. coli*, and ≈44.7 mm vs. *Aspergillus niger* [46]. Several studies have shown that combining chitosan with halloysite nanotubes creates a nanocomposite that often exhibits superior antibacterial properties compared to chitosan alone [47,48,49]. The results of our study clearly showed that the chitosan–HNT (**CS@HNT-*f***) composite showed higher antibacterial activity compared to the pure chitosan film. The enhanced antibacterial action of the chitosan–halloysite nanotube (HNT) composite is generally attributed to a combination of electrostatic interactions, structural effects, and improved availability of active groups [50]. Chitosan’s positively charged amino groups can bind to negatively charged bacterial cell surfaces, disrupting membrane integrity and increasing permeability [51]. When HNTs are incorporated, their high surface area and tubular structure promote interaction with bacterial cells [52]. Additionally, HNTs may facilitate a more uniform distribution and exposure of chitosan’s active sites, leading to more efficient interaction with microbial membranes. The mechanism of antibacterial action of CS-HNT films enhances bacterial membrane disintegration, disrupts cellular metabolism, and ultimately inhibits bacterial growth more effectively than chitosan alone.

Hemolysis and hemoglobin adsorption results suggest that while chitosan’s amino groups facilitate protein binding, excessive adsorption may underestimate hemolysis levels. Hemoglobin released from lysed erythrocytes can bind to the film surface rather than remaining in the supernatant, resulting in lower absorbance readings during spectrophotometric analysis. Consequently, the actual extent of hemolysis induced by the tested materials may be higher than indicated by conventional methods. This phenomenon is consistent with previous reports describing the strong affinity of chitosan for blood components, particularly proteins such as hemoglobin [42].

The hemoglobin adsorption capacity of chitosan-based films is strongly influenced by their chemical structure. Chitosan’s amino groups play a central role in protein binding, facilitating interactions with hemoglobin and other biomolecules. Chemical modifications that block or alter these functional groups, such as crosslinking or incorporation of bulky substituents, can significantly reduce the material’s ability to bind hemoglobin. This tunable adsorption behavior is particularly relevant in the design of chitosan-based materials for biomedical and environmental applications, such as hemostatic dressings, filtration systems and packaging materials, where controlled protein interaction is essential [53]. These findings emphasize the need for careful interpretation of hemolysis data when evaluating the blood compatibility of chitosan-based biomaterials, especially in applications where accurate assessment of erythrocyte integrity is critical.

Cytotoxicity assays confirmed the biocompatibility of most films, with CS-MMT and CS–sepiolite potentially promoting cell proliferation. Slight reductions in viability for CS-HNT warrant further optimization.

Overall, the majority of chitosan-based films tested were well tolerated by BJ fibroblasts, supporting their potential use in skin-contact applications. However, the reduced viability observed in select samples highlights the importance of careful material design and screening for cytotoxic effects.

There are several papers in the literature describing the degradation of pure chitosan membranes [54,55,56,57]. Unfortunately, although the biopolymer material itself is biodegradable, its enrichment with active compounds may disturb the biodegradation process by natural soil microflora. Therefore, one of the aims of the presented work was to assess the impact of the filler on the biodegradability of composites in the soil environment. Due to the current environmental pollution, films that can potentially be used in the medical or packaging industry must be completely biodegradable.

The most desirable effect of our research was to obtain a composite variant with excellent mechanical and physicochemical properties, high antimicrobial potential and no cytotoxicity. Given the growing role of chitosan in sectors such as food packaging, medicine, cosmetics and agriculture, it is extremely important to prove that chitosan films are safe for humans. However, the future of sustainable materials depends not only on their safety for human health but also on their environmental friendliness. Replacing environmentally harmful synthetic polymers with biopolymers is highly desirable and is expected to play a central role in strategies aimed at eliminating the risks posed by micro- and nanoplastics. Therefore, it is important to assess the biodegradability of chitosan-based films. Literature data describe the chitosan degradation process as several stages leading to the disintegration of polymer chains. The first stage is the swelling of the membranes initiated by water. This hydration step facilitates the diffusion of enzymes and microorganisms into the film matrix. Enzymes such as chitosanase, lysozyme, and proteases target the glycosidic bonds in chitosan, breaking them down into smaller fragments. The initial stage of the proposed chitosan degradation mechanism involves depolymerization, characterized by the random cleavage of β-1,4-glycosidic bonds, followed by deacetylation through hydrolysis of N–acetyl bonds. As a consequence of these processes, the degree of deacetylation increases and the molecular weight decreases [58]. The cleavage of chitosan functional groups (amine, carbonyl, amide and hydroxyl) may occur simultaneously, depending on chemical and/or enzymatic conditions. Chitosanase is a key enzyme in the initial stages of degradation, cleaving chitosan chains at random points [59]. Thanks to enzymatic hydrolysis, the chitosan film is gradually broken down into oligosaccharides and ultimately into single monomers such as glucosamine (D-glucosamine or N-acetylglucosamine), which can be further used by microorganisms [58]. We observed the fastest degradation of chitosan (**CS-*f***), chitosan–chitin (**CS@ChitMC-*f***), and chitosan–MMT (**CS@MMT-*f***) films. Films with the addition of sepiolite and halloysite nanotubes remained in the soil environment longer. A study analyzing the degradation of chitosan films in commercial composts, garden soil, and vineyard soil showed that after 10 days, the chitosan films were completely degraded [60]. Milbreta et al. (2024) showed that chitosan or alginate films with the addition of nanofibrillated cellulose were completely decomposed within 3 weeks in the soil environment [61]. The high cross-linking capacity of chitosan films with mineral clays contributed to their greater stability and resistance to biodegradation factors present in the soil. However, after 6 weeks of incubation, 100% biodegradability was observed.

Taking into account the acquisition of a stable composite with high antibacterial activity, non-cytotoxic and completely biodegradable in the soil environment after 4 weeks, films CS-MMT and CS–sepiolite with high potential for industrial use could be selected. These characteristics make them strong candidates for applications in packaging, medicine, and other sustainable material technologies

## 5. Conclusions

The results of this study confirm that the incorporation of specific fillers into chitosan films can significantly influence their biological activity and biodegradation profile. Among the tested nanocomposites, films such as CS–montmorillonite (***CS@MMT-f***) and CS–sepiolite (***CS@Sep-f***) demonstrated a well-balanced combination of desirable properties, including strong antibacterial activity, non-cytotoxicity, and complete biodegradability within 4 to 6 weeks in a soil environment. Notably, ***CS@MMT-f*** film exhibited the highest antibacterial effectiveness against *Staphylococcus aureus* and *Escherichia coli*. These findings highlight the potential of chitosan-based nanocomposite films as sustainable alternatives to conventional plastic materials, particularly in applications where both antimicrobial performance and environmental safety are essential.

## Figures and Tables

**Table 1:** Chemical composition of filled chitosan nanocomposite films.

Composition	Code
Chitosan	**CS-*f***
Chitosan–Chitine	**CS@ChitMC-*f***
Chitosan–MMT	**CS@MMT-*f***
Chitosan–Sepiolite	**CS@Sep-*f***
Chitosan–HNT	**CS@HNT-*f***

## Data Availability

The original contributions presented in this study are included in the article/Supplementary Material. Further inquiries can be directed to the corresponding author.

## Supplementary Materials

The following supporting information can be downloaded at https://www.mdpi.com/article/10.3390/ma19102167/s1, Table S1: Selected types of fillers in the chitosan matrix and their mechanisms of action [41,62,63,64,65,66,67,68,69,70,71,72,73,74,75,76,77,78,79,80,81,82,83,84,85,86,87,88,89,90,91,92,93].
