# Supplementary material for: Green Chitosan Bioplastics: How the Filler Impacts the Biological Activity and the Biodegradability?
Source: Materials (Basel). 2026 May 21;19(10):2167. doi: 10.3390/ma19102167 (PMC13208214; doi:10.3390/ma19102167)
Supplement: Supplementary file 1 [file materials-19-02167-s001.zip › materials-4310266-supplementary.pdf]

Table S1. Selected types of fillers in the chitosan matrix and their mechanisms of action.

Type of fillers	Improvement of mechanical properties	Mechanism of enhanced antimicrobial activity	Ref.
	**Carbon-based nanomaterials**		
**Carbon nanotubes (CNTs)**	Load transfer (increased tensile strength and modulus); energy dissipation (improved impact resistance); crack deflection (increased fracture toughness); polymer chain confinement (increased stiffness and dimensional stability); improvement of the mechanical properties of CS nanocomposites; increased of strength, stiffness and elasticity of coatings.	Physical disruption of cell membranes (leads to leakage of intracellular contents and cell lysis); ROS generation (oxidative stress, DNA damage); extraction of phospholipids (membrane destabilization); genotoxicity and DNA damage (inhibition of replication); electron transport disruption; inhibition of biofilm formation (prevents microbial adhesion).	62-64
**Carbon quantum dots (CQDs)**	Nanofiller reinforcement (enhanced toughness, tensile strength, and resistance to micro-cracks); interfacial bonding (improved interfacial adhesion and toughness); chain immobilization (higher thermal stability and modulus); crosslinking (enhanced elasticity, stiffness, and stretchability).	ROS generation (oxidative stress, DNA damage); electrostatic interaction with cell membranes (increased permeability of bacterial membranes and leakage of intracellular contents); genotoxicity and DNA damage (inhibition of replication); disruption of metabolic activity (enzyme inhibition); photothermal effect; inhibition of biofilm formation (prevents microbial adhesion).	65-66
**Graphene oxide (GO)**	2D filler reinforcement (increased modulus, crack resistance); interfacial bonding (higher tensile strength and modulus); crosslinking (increased elasticity, stretchability, and mechanical durability); polymer chain confinement (higher stiffness and thermal stability); crack deflection (increased fracture toughness)	physical disruption of cell membranes (leads to leakage of intracellular contents and cell lysis); ROS generation (oxidative stress, DNA damage); inhibition of biofilm formation (prevents microbial adhesion).	41, 67-68
	**Metal and metal oxide nanoparticles**		
**AgNPs**	Improve stress transfer between matrix and filler; increased tensile strength and modulus; enhanced interfacial adhesion; crack deflection (increased fracture toughness).	Release Ag^{+} ions that increase membrane permeability; ROS generation (oxidative stress, DNA damage); DNA/protein interaction; inhibition of biofilm formation (prevents microbial adhesion).	69-71

**ZnO**	Filler reinforcement (increases tensile strength and stiffness); interfacial bonding (enhanced toughness); crack deflection (improves fracture resistance); uniform dispersion (prevents aggregation).	ROS generation (oxidative stress, DNA damage); cell membrane destabilization and increased permeability; interaction with intracellular components; photocatalytic activity (enhanced ROS production).	72-73
**TiO_2**	Filler reinforcement (increases tensile strength and stiffness); interfacial bonding (enhanced toughness); crack deflection (improves fracture resistance); uniform dispersion (prevents aggregation).	ROS generation (oxidative stress, DNA damage); cell membrane destabilization and increased permeability; interaction with intracellular components; inhibition of biofilm formation (prevent microbial adhesion).	74-75
	**Clay and Bioceramic Nanoparticles**		
**Montmorillonite (MMT)**	Nanofiller reinforcement (increases strength and stiffness); interfacial bonding (enhanced toughness); high aspect ratio and layered structure (enhance modulus with minimal filler content); crack deflection (improves fracture toughness); intercalation and exfoliation structures (improvedmechanical properties).	Surface interactions (adsorbs and disrupts microbial membranes); ion exchange and release of antimicrobial agents; drug delivery (protects the antimicrobial agents, drug dose reduction); nutrient absorption (inhibits microbial growth indirectly).	76-77
**Halloysite nanotubes (HNTs)**	Materials reinforcement through load transfer; crack deflection (improves fracture toughness); improved interfacial adhesion (prevents interfacial debonding); barrier effect (increased dimensional and thermal stability); homogeneous dispersion (enhanced mechanical integrity).	Surface interactions (adsorbs and disrupts microbial membranes); antibiofilm activity (improved antimicrobial drug penetration); controlled release of antimicrobial drugs (prolonged antimicrobial activity, drug dose reduction); photocatalysis (with TiO_2, ZnO) (ROS production upon light exposure).	78-81
**Sepiolite**	Fibrous morphology (increased tensile strength, fracture toughness of nanocomposites); interfacial bonding (surface -OH groups interact with the matrix, which improved stress transfer efficiency; prevents filler slippage); thixotropic and rheological behaviour (structural stability); stress absorption and energy dissipation (due to its tunnel-like structure); functionalizability (enhanced compatibility and	Surface interactions (adsorbs and disrupts microbial membranes); drug carrier (controlled release and prolonged antimicrobial effect); nanoparticle stabilization (prevents aggregation in composites); barrier effect (indirectly inhibits microbial growth).	82-83

	dispersion).		
**Hydroxyapatite (HAP)**	Load bearing (increased stiffness and tensile/compressive strength); crack deflection (increased fracture toughness); interfacial bonding (e.g., –OH, PO_4^{3-}, Ca^{2+}groups interact with organic matrices or biointerfaces, which improves stress transfer efficiency); biomimetic reinforcement (enhanced long-term mechanical strength); nano-scale effect – nano-HAP (higher reinforcement with lower filler content).	Surface interactions (adsorbs and disrupts microbial membranes); ion release (Ca^{2+}, PO_4^{3-}) causes metabolic disruption in microorganisms; nanoparticle size: nanoHAP (improved surface contact and penetration); antibiofilm activity (reduced adhesion and colonization of bacteria)	84-85
	**Natural nanofillers**		
**Strach nanoparticles**	Filler reinforcement (increased tensile strength and Young's modulus); interfacial bonding (surface -OH groups interact with the matrix, which improves stress transfer efficiency, prevents filler slippage); crack deflection (improved fracture toughness and durability); crystallinity enhancement (increased modulus and dimensional stability); network formation and dispersion (even load distribution, reduced brittleness).	Drug carrier (controlled release and prolonged antimicrobial effect); nanoparticle stabilization (prevents aggregation in composites); antibiofilm activity (reduced adhesion and colonization of bacteria); controlled release (prolonged antimicrobial effect).	86-88
**Nanocellulose**	Interfacial adhesion (better stress transfer, elongation, toughness); high aspect ratio and intrinsic strength (improved tensile strength and stiffness); filler reinforcement and crystallinity (increased modulus and dimensional stability); network formation and percolation (fracture resistance, uniform stress distribution); barrier to polymer mobility (improved thermal and mechanical stability).	Drug carrier (controlled release); electrostatic interactions (increased permeability of bacterial membranes and leakage of intracellular contents); physical membrane disruption (high-aspect-ratio nanocellulose forms may mechanically damage cell walls); biofilm prevention (modulates hydration and prevents bacterial colonization).	89-90
**Chitin nanowhiskers**	Interfacial bonding (improved toughness and dimensional stability); crystallinity (High stiffness and strength); high aspect ratio (better reinforcement and crack-bridging capability); network formation and percolation (increased mechanical integrity);	Electrostatic interactions with bacterial membranes (increased permeability of membranes and leakage of intracellular contents); physical disruption of cell membranes (needle-like morphology causes mechanical damage to membranes);	91-93

	polymer chain mobility restriction (improved mechanical durability and thermal stability).	antibiofilm activity (reduced adhesion and colonization of bacteria); metal ion delivery (enhances antimicrobial action).	

41. Wrońska, N.; Anouar, A.; El Achaby, M.; Zawadzka, K.; Kędzierska, M.; Miłowska, K.; Katir, N.; Draoui, K.; Różalska, S.; Piwoński, I.; et al. Chitosan-Functionalized Graphene Nanocomposite Films: Interfacial Interplay and Biological Activity. *Materials* **2020**, *13*, 998. https://doi.org/10.3390/ma13040998.

62. Lessa, E.F.; Gerhardt, R.; Arabidian, V.; Da Silva, K.A.; Da Silveira Junior, N.; Jaeschke, D.P.; Cadaval Junior, T.R.S.; Pinto, L.A.D.A. Synthesis of Chitosan/Carbon Nanotubes Composite Films as Potential Removal of Anionic and Cationic Dyes in Aqueous Solutions. *ACS Omega* **2025**, *10*, 31840–31853. https://doi.org/10.1021/acsomega.5c03164.
63. Demirel, A.; Yılmaz, E.; Türk, S.; Çalışkan, F. Preparation and Investigation of Porous Chitosan/Carbon Nanotube Biocomposite Coating by Space Holder Method. *Diam. Relat. Mater.* **2023**, *138*, 110217. https://doi.org/10.1016/j.diamond.2023.110217.
64. Magallanes-Vallejo, A.G.; López-Oyama, A.B.; González, E.R.; Del Angel-López, D.; Pulido-Barragán, E.U.; García-Guendulain, C.; Madera-Santana, T.J.; Rodríguez-Beas, C.; Gámez-Corrales, R. Study of the Influence of Chitosan-Wrapped Carbon Nanotubes on Biopolymer Film Properties. *Polymers* **2025**, *1*, 889. https://doi.org/10.3390/polym17070889.
65. Wen, F.; Li, P.; Yan, H.; Su, W. Turmeric Carbon Quantum Dots Enhanced Chitosan Nanocomposite Films Based on Photodynamic Inactivation Technology for Antibacterial Food Packaging. *Carbohydr. Polym.* **2023**, *311*, 120784. https://doi.org/10.1016/j.carbpol.2023.120784.
66. Fu, B.; Liu, Q.; Liu, M.; Chen, X.; Lin, H.; Zheng, Z.; Zhu, J.; Dai, C.; Dong, X.; Yang, D.-P. Carbon Dots Enhanced Gelatin/Chitosan Bio-Nanocomposite Packaging Film for Perishable Foods. *Chin. Chem. Lett.* **2022**, *33*, 4577–4582. https://doi.org/10.1016/j.cclet.2022.03.048.
67. Jin, L.; Chen, Q.; Hu, X.; Chen, H.; Lu, Y.; Zhang, Y.; Zhou, H.; Bai, Y. Enhanced Mechanical Strength and Antibacterial Properties of Chitosan/Graphene Oxide Composite Fibres. *Cellulose* **2022**, *29*, 3889–3900. https://doi.org/10.1007/s10570-022-04523-8.
68. Ramezani Farani, M.; Zare, I.; Mirshafiei, M.; Gholami, A.; Zhang, M.; Pishbin, E.; Ahn, J.E.; Mohammadi, A.; Imani, M.; Lak, M.; et al. Graphene Oxide-Engineered Chitosan Nanoparticles: Synthesis, Properties, and Antibacterial Activity for Tissue Engineering and Regenerative Medicine. *Chem. Eng. J.* **2025**, *509*, 160852. https://doi.org/10.1016/j.cej.2025.160852.
69. Harun-Ur-Rashid, M.; Foyez, T.; Krishna, S.B.N.; Poda, S.; Imran, A.B. Recent Advances of Silver Nanoparticle-Based Polymer Nanocomposites for Biomedical Applications. *RSC Adv.* **2025**, *15*, 8480–8505. https://doi.org/10.1039/D4RA08220F.
70. Ali, S.; Bahadur, A.; Hassan, A.; Ahmad, S.; Shah, W.; Iqbal, S. Optimized Silver Nanostructures for Enhanced Antibacterial Potential: Recent Trends and Challenges in the Development of Metallo-Antimicrobials. *Chem. Eng. J.* **2025**, *507*, 160470. https://doi.org/10.1016/j.cej.2025.160470.
71. Tripathi, N.; Goshisht, M.K. Recent Advances and Mechanistic Insights into Antibacterial Activity, Antibiofilm Activity, and Cytotoxicity of Silver Nanoparticles. *ACS Appl. Bio Mater.* **2022**, *5*, 1391–1463. https://doi.org/10.1021/acsabm.2c00014.
72. Mujeeb Rahman, P.; Abdul Mujeeb, V.M.; Muraleedharan, K.; Thomas, S.K. Chitosan/Nano ZnO Composite Films: Enhanced Mechanical, Antimicrobial and Dielectric Properties. *Arab. J. Chem.* **2018**, *11*, 120–127. https://doi.org/10.1016/j.arabjc.2016.09.008.
73. Wang, Y.; Liu, J.; Wang, T.; Liu, L.-Z.; Tian, C.; Cui, Y.; Shao, W.; Hua, X.; Shi, Y.; Wang, Y. Antibacterial Properties and Mechanism of Nanometer Zinc Oxide Composites. *Food Packag. Shelf Life* **2023**, *40*, 101167. https://doi.org/10.1016/j.fpsl.2023.101167.
74. Winnicki, M.; Łatka, L.; Jasiorski, M.; Baszczuk, A. Mechanical Properties of TiO2 Coatings Deposited by Low Pressure Cold Spraying. *Surf. Coat. Technol.* **2021**, *405*, 126516. https://doi.org/10.1016/j.surfcoat.2020.126516.

75. Serov, D.A.; Gritsaeva, A.V.; Yanbaev, F.M.; Simakin, A.V.; Gudkov, S.V. Review of Antimicrobial Properties of Titanium Dioxide Nanoparticles. *Int. J. Mol. Sci.* **2024**, *25*, 10519. https://doi.org/10.3390/ijms251910519.
76. Jemai, R.; Chalghaf, R.; Boubakri, S.; Amine Djebbi, M.; Naamen, S.; Ben Rhaiem, H.; Ben Haj Amara, A. Montmorillonite: Properties, Characteristics, and Its Harnessing in Environmental Applications. In *Recent Advances in Montmorillonite*; Morari Do Nascimento, G., Ed.; IntechOpen: London, UK, 2024. https://doi.org/10.5772/intechopen.1004763.
77. Qian, Y.; Huang, Z.; Zhou, G.; Chen, C.; Sang, Y.; Yu, Z.; Jiang, L.; Mei, Y.; Wei, Y. Preparation and Properties of Organically Modified Na-Montmorillonite. *Materials* **2023**, *16*, 3184. https://doi.org/10.3390/ma16083184.
78. Gao, H.; Soto, M.A.; Szymkowiak, J.K.; Andrew, L.J.; Hamad, W.Y.; MacLachlan, M.J. Halloysite Nanotubes Enhance the Mechanical Properties and Thermal Stability of Iridescent Cellulose Nanocrystal Films. *Dalton Trans.* **2023**, *52*, 7136–7142. https://doi.org/10.1039/D3DT00498H.
79. Dashti Rahmatabadi, M.; Eslami, H.; Shafiei, S.S.; Ansari, M. Biological and Mechanical Effects of Halloysite Nanotubes and Erythropoietin Release in Brushite-Based Bone Cements. *Mater. Chem. Phys.* **2025**, *346*, 131371. https://doi.org/10.1016/j.matchemphys.2025.131371.
80. Biddeci, G.; Spinelli, G.; Colomba, P.; Di Blasi, F. Nanomaterials: A Review about Halloysite Nanotubes, Properties, and Application in the Biological Field. *Int. J. Mol. Sci.* **2022**, *23*, 11518. https://doi.org/10.3390/ijms231911518.
81. Biddeci, G.; Spinelli, G.; Colomba, P.; Di Blasi, F. Halloysite Nanotubes and Sepiolite for Health Applications. *Int. J. Mol. Sci.* **2023**, *24*, 4801. https://doi.org/10.3390/ijms24054801.
82. Jiang, Y.; Wang, L.; Qi, W.; Yin, P.; Liao, X.; Luo, Y.; Ding, Y. Antibacterial and Self-Healing Sepiolite-Based Hybrid Hydrogel for Hemostasis and Wound Healing. *Biomater. Adv.* **2024**, *159*, 213838. https://doi.org/10.1016/j.bioadv.2024.213838.
83. Iqbal Khan, Z.; Habib, U.; Binti Mohamad, Z.; Razak Bin Rahmat, A.; Amira Sahirah Binti Abdullah, N. Mechanical and Thermal Properties of Sepiolite Strengthened Thermoplastic Polymer Nanocomposites: A Comprehensive Review. *Alex. Eng. J.* **2022**, *61*, 975–990. https://doi.org/10.1016/j.aej.2021.06.015.
84. Hassanain, M.; Abdel-Ghafar, H.M.; Hamouda, H.I.; El-Hosiny, F.I.; Ewais, E.M.M. Enhanced Antimicrobial Efficacy of Hydroxyapatite-Based Composites for Healthcare Applications. *Sci Rep* **2024**, *14*, 26426. https://doi.org/10.1038/s41598-024-76088-4.
85. Mondal, S.; Park, S.; Choi, J.; Vu, T.T.H.; Doan, V.H.M.; Vo, T.T.; Lee, B.; Oh, J. Hydroxyapatite: A Journey from Biomaterials to Advanced Functional Materials. *Adv. Colloid Interface Sci.* **2023**, *321*, 103013. https://doi.org/10.1016/j.cis.2023.103013.
86. Marta, H.; Rizki, D.I.; Mardawati, E.; Djali, M.; Mohammad, M.; Cahyana, Y. Starch Nanoparticles: Preparation, Properties and Applications. *Polymers* **2023**, *15*, 1167. https://doi.org/10.3390/polym15051167.
87. Hou, X.; Wang, H.; Shi, Y.; Yue, Z. Recent Advances of Antibacterial Starch-Based Materials. *Carbohydr. Polym.* **2023**, *302*, 120392. https://doi.org/10.1016/j.carbpol.2022.120392.
88. Shi, A.; Wang, L.; Li, D.; Adhikari, B. Characterization of Starch Films Containing Starch Nanoparticles. *Carbohydr. Polym.* **2013**, *96*, 593–601. https://doi.org/10.1016/j.carbpol.2012.12.042.
89. Norrrahim, M.N.F.; Nurazzi, N.M.; Jenol, M.A.; Farid, M.A.A.; Janudin, N.; Ujang, F.A.; Yasim-Anuar, T.A.T.; Syed Najmuddin, S.U.F.; Ilyas, R.A. Emerging Development of Nanocellulose as an Antimicrobial Material: An Overview. *Mater. Adv.* **2021**, *2*, 3538–3551. https://doi.org/10.1039/D1MA00116G.
90. Deng, N.; Li, Q.; Wang, W. Design and Fabrication of Nanocellulose-Chitosan Composite Hydrogels with Enhanced Mechanical and Antibacterial Properties. *Langmuir* **2025**, *41*, 13604–13610. https://doi.org/10.1021/acs.langmuir.5c01538.
91. Panackal Shibu, R.; Jafari, M.; Sagala, S.L.; Shamshina, J.L. Chitin Nanowhiskers: A Review of Manufacturing, Processing, and the Influence of Content on Composite Reinforcement and Property Enhancement. *RSC Appl. Polym.* **2025**, *3*, 1031–1123. https://doi.org/10.1039/D5LP00104H.

92. Qin, Y.; Zhang, S.; Yu, J.; Yang, J.; Xiong, L.; Sun, Q. Effects of Chitin Nano-Whiskers on the Antibacterial and Physicochemical Properties of Maize Starch Films. *Carbohydr. Polym.* **2016**, *147*, 372–378. https://doi.org/10.1016/j.carbpol.2016.03.095.
93. Abdelrahman, R.M.; Abdel-Mohsen, A.M.; Zboncak, M.; Frankova, J.; Lepcio, P.; Kobera, L.; Steinhart, M.; Pavlinak, D.; Spotaz, Z.; Sklenářévá, R.; et al. Hyaluronan Biofilms Reinforced with Partially Deacetylated Chitin Nanowhiskers: Extraction, Fabrication, in-Vitro and Antibacterial Properties of Advanced Nanocomposites. *Carbohydr. Polym.* **2020**, *235*, 115951. https://doi.org/10.1016/j.carbpol.2020.115951.
